# Supplementary material for: Science of music-based citizen science: How seeing influences hearing
Source: PLoS One. 2025 Sep 10;20(9):e0325019. doi: 10.1371/journal.pone.0325019 (PMC12422445; doi:10.1371/journal.pone.0325019)

Science of music-based citizen science: How seeing influences hearing

Daniel Bedoya, Paul Lascabettes, Lawrence Fyfe, Elaine Chew

Supporting information: S2 File. Annotation Instructions

The following text contains the English transcription of the instructions for the cross-modal annotations experiment, as presented to the participants.

1 Part one

The first part of the instructions explains the task and the definition of boundaries that was used for the experiment:

Experiment annotation instructions - part 1

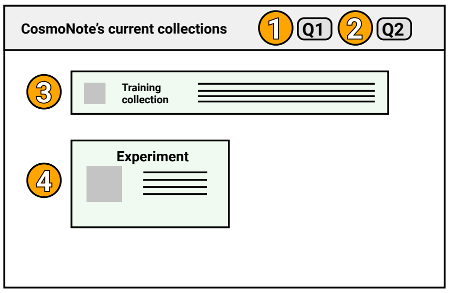

What are you asked to do?

1. Fill in a Short Musical Questionnaire [3 min].
2. Calibrate your sound and do a Hearing environment test [2 min].
3. Familiarize with the interface on the Training Collection (your answers won't be saved) [5 min].
4. Main Task [∼85 min]: Please **mark the boundaries** that you hear in the music and **indicate the strength** of each boundary. You may be presented with visual information layers such as a waveform or notes. **Your ear should be your main guide** for the pieces where sound is available.

When you finish all the pieces, you'll be asked to fill a feedback questionnaire [5 min].

What is a boundary?

Boundaries are time points that separate a music stream into segments representing meaningful chunks of music e.g., a musical idea or a musical thought. Boundaries not only separate a larger piece of music into smaller, coherent units, they also help listeners make sense of the music.

There are four levels of boundaries, defined from 1 (weakest) to 4 (strongest).

How do performers communicate boundaries?

Performers may mark boundaries using pauses, stress, or contrast. For example, accents could mark the beginnings of groups of notes, pauses can separate musical ideas, phrases may be expressed by increasing then decreasing tempo and/or loudness, a change of timbre and loudness may mark the beginning of a new section.

---

2 Part two

The second part of the instructions explains the three possible interface configurations of the CosmoNote interface, provides a few technical instructions to place boundaries in CosmoNote, and mentions common annotation tips:

Experiment annotation instructions - part 2

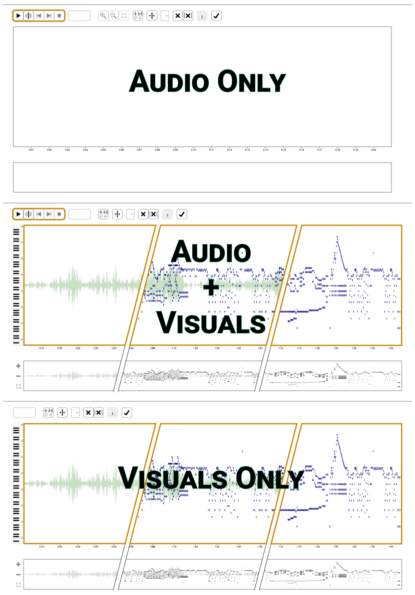

What you will see/hear

In this experiment, you will annotate 33 pieces of music, shown in different configurations of the CosmoNote interface.
You'll be presented with one of three cases:

1. *Audio only*: Listen to the music to annotate. When you press play (or spacebar) you'll see the playhead position in time but won't see any other visual representations.
2. *Audio + visuals*: Listen to the music to annotate. When you press play (or spacebar) you'll see the playhead position in time. You may use visual representations to help you annotate.
3. *Visuals only*: Annotate visually. You'll see visual representations of the music but won't be able to play/hear any sound.

How to place boundaries

There are two ways to place a boundary:

1. While the audio is stopped (press) : Select the boundary button) and click anywhere on the screen; you can select the boundary strength level from the dropdown list next to the boundary button.
2. While the audio is playing (): Press [1], [2], [3] or [4] on your keyboard (the strength is defined by the key you press).

You can click on boundaries to adjust them (level/position) if necessary.

Remember to save often and push on the "Finish" button in order to advance to the next piece.

Tips

- Don't dwell on the same piece for too long.
- Feel free to take small breaks every 10 pieces.

The following image shows a common way of annotating in CosmoNote:

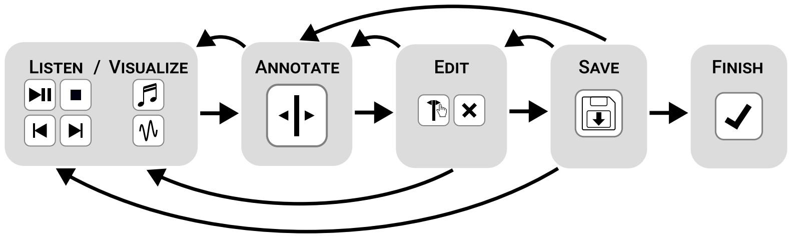

Supplement: S2 File — Document with the instructions given to participants during the study. (PDF) [file pone.0325019.s001.pdf]
