# Supplementary material for: Science of music-based citizen science: How seeing influences hearing
Source: PLoS One. 2025 Sep 10;20(9):e0325019. doi: 10.1371/journal.pone.0325019 (PMC12422445; doi:10.1371/journal.pone.0325019)
Supplement: S1 Table — Table with smallest uOT distances between all comparisons. (PDF) [file pone.0325019.s002.pdf]

Science of music-based citizen science: How seeing influences hearing

Daniel Bedoya, Paul Lascabettes, Lawrence Fyfe, Elaine Chew

Supporting information: S1 Table. Smallest distances

The following table contain rankings of the explicit values of unbalanced optimal transport distances between the grouped conditions defined in the experiment. The uOT normalized column shows the distance values scaled by the duration of each piece. For this ranking, we start by considering all pieces and comparisons equally. That is, 231 points from 33 pieces and 7 comparisons. Then, we keep only the smallest distances calculated for each piece.

Title	Duration	uOT	uOT normalized	Comparison
Var XXXII	129.04	64.04	0.5	AV vs. A
Var XIII	20.75	11.6	0.56	AV vs. V
Var XXX	25.45	14.7	0.58	AV vs. V
Var XXXI	28.52	16.79	0.59	AV vs. V
Var XXVII	18.76	11.25	0.6	AV vs. V
Tema	18.74	11.36	0.61	AV vs. V
Var III	14.55	8.91	0.61	AV vs. V
Var XXIV	14.25	8.82	0.62	AV vs. V
Var XVIII	24.75	15.49	0.63	AV vs. V
Var XVI	18.74	11.77	0.63	AV vs. V
Var XXI	13.26	8.72	0.66	AV vs. V
Var XXVI	15.68	10.55	0.67	V vs. A
Var XII	35.01	23.64	0.68	AV vs. V
Var XIX	13.56	9.32	0.69	AV vs. V
Var V	18.03	12.84	0.71	AV vs. V
Var IX	24.27	17.71	0.73	AV vs. V
Var VIII	19.47	14.76	0.76	AV vs. V
Var XXV	13.86	10.65	0.77	AV vs. V
Var XX	12.59	9.74	0.77	AV vs. P
Var IV	16.97	13.26	0.78	AV vs. V
Var X	16.84	13.2	0.78	AV vs. V
Var XXVIII	22.35	17.56	0.79	AV vs. V
Var II	13.22	10.57	0.8	AV vs. V
Var XI	16.42	14.5	0.88	AV vs. W
Var XIV	21.89	19.43	0.89	AV vs. V
Var XXIX	16.1	14.7	0.91	AV vs. V
Var I	13.79	12.6	0.91	AV vs. V
Var XXIII	16.92	15.9	0.94	AV vs. V
Var VI	15.4	14.56	0.95	AV vs. V
Var XVII	22.22	21.69	0.98	AV vs. V
Var XV	18.91	18.97	1	AV vs. A
Var VII	20.82	20.93	1.01	AV vs. V
Var XXII	15.37	17.2	1.12	AV vs. A

Table 1: Smallest uOT distances between visual and aural annotations in all pieces of Beethoven's 32 Variations in C minor. Exceptions to the trend are shown in red.
