# Supplementary material for: Science of music-based citizen science: How seeing influences hearing
Source: PLoS One. 2025 Sep 10;20(9):e0325019. doi: 10.1371/journal.pone.0325019 (PMC12422445; doi:10.1371/journal.pone.0325019)
Supplement: S2 Table — Table with largest uOT distances between all comparisons. (PDF) [file pone.0325019.s003.pdf]

Science of music-based citizen science: How seeing influences hearing

Daniel Bedoya, Paul Lascabettes, Lawrence Fyfe, Elaine Chew

Supporting information: S2 Table. Largest distances

The following table contain rankings of the explicit values of unbalanced optimal transport distances between the grouped conditions defined in the experiment. The uOT normalized column shows the distance values scaled by the duration of each piece. For this ranking, we start by considering all pieces and comparisons equally. That is, 231 points from 33 pieces and 7 comparisons. Then, we keep only the largest distances calculated for each piece.

Title	Duration	uOT	uOT normalized	Comparison
Var XXXII	129.04	162.05	1.26	W vs. A
Var XVIII	24.75	31.92	1.29	P vs. A
Var XXVI	15.68	22.5	1.43	AV vs. P
Var IV	16.97	26.13	1.54	P vs. A
Var XXV	13.86	21.95	1.58	W vs. A
Var XIX	13.56	22.03	1.62	P vs. A
Var XIII	20.75	34.08	1.64	W vs. A
Var XXII	15.37	25.64	1.67	W vs. A
Var XI	16.42	28.24	1.72	P vs. A
Var XII	35.01	60.56	1.73	P vs. A
Var I	13.79	24.44	1.77	W vs. A
Var XXXI	28.52	51.92	1.82	AV vs. W
Var V	18.03	36.18	2.01	P vs. A
Var XXIV	14.25	29.79	2.09	W vs. A
Var XXVII	18.76	40.49	2.16	W vs. A
Var XXX	25.45	56.6	2.22	P vs. A
Var XXVIII	22.35	50.92	2.28	W vs. A
Var XX	12.59	29.18	2.32	W vs. A
Var XXI	13.26	31.25	2.36	W vs. A
Var XVI	18.74	44.36	2.37	P vs. A
Var III	14.55	34.51	2.37	P vs. A
Var X	16.84	40.57	2.41	W vs. A
Var IX	24.27	62.36	2.57	P vs. A
Var XVII	22.22	57.26	2.58	W vs. A
Var VII	20.82	53.77	2.58	W vs. A
Var VIII	19.47	50.33	2.59	W vs. A
Var XV	18.91	50.46	2.67	P vs. A
Var XXIII	16.92	46.54	2.75	P vs. A
Var VI	15.4	43.58	2.83	W vs. A
Var II	13.22	37.77	2.86	P vs. A
Var XXIX	16.1	46.46	2.89	W vs. A
Tema	18.74	61.93	3.3	P vs. A
Var XIV	21.89	83.04	3.79	P vs. A

Table 1: Largest uOT distances between visual and aural annotations in all pieces of Beethoven's 32 Variations in C minor. Exceptions to the trend are shown in red.
