# Supplementary material for: Science of music-based citizen science: How seeing influences hearing
Source: PLoS One. 2025 Sep 10;20(9):e0325019. doi: 10.1371/journal.pone.0325019 (PMC12422445; doi:10.1371/journal.pone.0325019)
Supplement: Attachment — Submitted filename: Response_to_reviewers.pdf [file pone.0325019.s004.pdf]

Thursday, May 1, 2025

Dear editor and reviewers,
PLOS ONE

Thank you for your time and for your comments regarding our article entitled "**Science of music-based citizen science: How seeing influences hearing**".

The following paragraphs contain a description of the changes we made to the manuscript. Line numbers refer to the original version of the manuscript for comparison.

Form (typos, missing notation, etc.)

- Rephrased unclear text on line 20.
- Corrected typos on lines: 39, 287.
- Clarified indices *k* and *n* form the expressions in Equation 2:

Optimal transport

Let f and g be two discrete functions from $\mathbb{N}$ with values in $\mathbb{R}^+$, representing two sets of weighted boundary annotations. The distribution function F of f, and $\|f\|_1$, the total sum of the boundaries of f, are defined in Eq 2 as follows:

$$F(n) = \sum_{k=0}^{n} f(k), \qquad \|f\|_1 = \sum_{k=0}^{\infty} f(k). \tag{2}$$

Here, n represents the upper limit of the summation for $F(n)$, which accumulates the values of $f(k)$ from $k = 0$ to $k = n$, while $\|f\|_1$ denotes the total sum of $f(k)$ over all indices $k \in \mathbb{N}$. Therefore, the distance associated with discrete optimal transport,

- Accepted suggestions on line 246.
- Added a reference to support the Earth mover's distance denomination.
- Included a number and a reference to the equation on line 276.
- Specified the constant *c,* at line 286.
- Added x-tick labels to the x-axis in Figure 5.
- Regenerated Figure 7. The numbers were removed, the dots were enlarged, their opacity increased, and the legend now contains a border to differentiate it from the points.

Content

Figure 5: What are the F_alpha and uOT value ranges?

The F-measure and its variants have a range of 0 to 1. The lowest possible value of the uOT distance is 0. However, its highest value depends on the length of the piece, as stated in the manuscript. Thus, exact values are difficult to provide.

Figure 7: Why MDS, and not PCA or TSNE?

PCA works best when multiple variables can be used to support the 2D projection. The principal components in the projection are those that best explain the variance in the data. We do not compare multiple variables in our dataset; thus, PCA is less useful in our study. While t-SNE is also a good option for dimensional scaling, it introduces unnecessary complexity (e.g., tuning

parameters such as perplexity, method, and early exaggeration). In addition, this technique tends to prioritize the distance between neighbors. We chose MDS because it would preserve the distances between all the points in the distribution as much as possible while being simple to implement.

Sincerely,

Daniel Bedoya

Corresponding author
daniel.bedoya@univ-lille.fr
